# Supplementary material for: Personal Protective Equipment Portraits Canada (PPC)–Humanization and surveying mask-wearing nationally
Source: PLoS One. 2024 Feb 23;19(2):e0298052. doi: 10.1371/journal.pone.0298052 (PMC10889603; doi:10.1371/journal.pone.0298052)
Supplement: S1 Table — (DOCX) [file pone.0298052.s002.docx]

**S2 Table. Response type, themes, and subthemes for the thematic analysis.**

| **Response Type** | **Themes** | **Subthemes** |
| --- | --- | --- |
| Positive | Humanization of HCW / Medical benefit | Alleviating/ Comforting |
|  | Setting | Approachability |
|  | Work environment / Colleague interactions | Colleague Interactions |
|  |  | Communication |
|  |  | Compliment picture |
|  |  | Connection |
|  |  | Conversation starter/Icebreaker |
|  |  | Encouragement of the project |
|  |  | Familiarity / Identification |
|  |  | Medical benefit |
|  |  | Self-confidence |
|  |  | Smile back / Appreciative patient |
|  |  | Spread interest among HCW |
| Negative | Barriers | Awkward/ Uncomfortable |
|  | Logistics | Burden |
|  | Setting | Did not like their picture |
|  |  | Has not used the portrait |
|  |  | No work with patients |
|  |  | Patients unconscious |
|  |  | Received a negative comment |
|  |  | Size problem |
|  |  | Unable to assess |
| Indifferent | Barriers | Colleague Interactions |
|  | Logistics | Communication |
|  | No difference | Familiarity with colleagues pre-pandemic |
|  | Setting | Has not used the portrait |
|  | Work environment / Colleague interactions | Indifferent |
|  |  | Inappropriate setting |
|  |  | No work with patients |
|  |  | Patients unconscious |
