# Supplementary material for: Personal Protective Equipment Portraits Canada (PPC)–Humanization and surveying mask-wearing nationally
Source: PLoS One. 2024 Feb 23;19(2):e0298052. doi: 10.1371/journal.pone.0298052 (PMC10889603; doi:10.1371/journal.pone.0298052)
Supplement: Attachment — Submitted filename: Response to Reviewers.docx [file pone.0298052.s004.docx]

Comments

1) **Include information that answers the following questions:**
   a. **Was it possible to establish any relationship between the feedback obtained in the research and the profession of the participants?**
   b. **Was there any relationship between this factor and the experience with patients and colleagues?**
2) **Include in the paper a summary of the research graphics, also listing the professions of the participants.**

This is a really interesting idea. We’ve investigated the relationship between professions and the feedback/experience with patients and colleagues and summarized these with the inclusion of Figure 4.

3) **Present the results of the thematic qualitative analysis in graphic form, highlighting the percentage of positive, negative and indifferent responses, and for each of them, the respective percentage of responses according to the themes and subthemes presented in Supplementary Table 2.**

Thank you for this suggestion as we agree presenting the thematic qualitative analysis in graphic form will be easier for readers to interpret.

We’ve done this and is presented in Figure 5.

4) **Present the statistical analysis of the data obtained to support the discussion.**

We are not sure what the reviewers mean by this as the survey data is qualitative in nature and we don’t believe a statistical analysis to determine if the PPE portraits made a significant difference can be done. We did add in our limitations “*associating a numerical value to the qualitative responses at survey creation*” would have allowed for such analyses.

Do the reviews have a statistical analysis in mind that they would like us to run on the data? Any recommendations would be much appreciated.

5) **Add the figures and tables to the body of the work, and discuss the data presented further, as they are still a little confusing.**

We have added the figures and tables to the text and have added discussion points to clarify and deepen the results presented.
